# Automated identification of urban substructure for comparative analysis

**DOI:** 10.1371/journal.pone.0245067

**Published:** 2021-01-14

**Authors:** Rohan L. Aras, Nicholas T. Ouellette, Rishee K. Jain

**Affiliations:** Department of Civil and Environmental Engineering, Stanford University, Stanford, CA, United States of America; Irstea, FRANCE

## Abstract

Neighborhoods are the building blocks of cities, and thus significantly impact urban planning from infrastructure deployment to service provisioning. However, existing definitions of neighborhoods are often ill suited for planning in both scale and pattern of aggregation. Here, we propose a generalized, scalable approach using topological data analysis to identify *barrier-enclosed* neighborhoods on multiple scales with implications for understanding social mixing within cities and the design of urban infrastructure. Our method requires no prior domain knowledge and uses only readily available building parcel information. Results from three American cities (Houston, New York, San Francisco) indicate that our method identifies neighborhoods consistent with historical approaches. Additionally, we uncover a consistent scale in all three cities at which physical isolation drives neighborhood emergence. However, our methods also reveal differences between these cities: Houston, although more disconnected on larger spatial scales than New York and San Francisco, is less disconnected at smaller scales.

## Introduction

As the world has rapidly urbanized over the last several decades [1], a growing majority of the global population is directly affected by the organization and design of urban functions. There are significant corresponding needs to develop a robust scientific understanding of these systems and improve our provisioning of infrastructure systems. One impediment to satisfying both of these goals is the subdivision of cities into objective and justifiable intra-urban spatial units. Such “neighborhoods” inform the deployment of resources and provisioning of services by governing agencies, ranging from sanitation to mobility to healthcare [2–5], both in current practice and active research. As an example, redevelopment of informal settlements is often currently achieved through masterplans of these services at the neighborhood scale [3]. Similarly, active research suggests that understanding the spatial scale (that is, the geographic extent) at which neighborhoods naturally form can productively inform the design, size, and type of technology for distributed energy resources [4,6].

However, the subdivisions currently used to inform this provisioning are often arbitrarily defined, outdated, or otherwise unclearly suited for the task [7]. At best, there remains large uncertainty about the optimality of plans or policies developed using the corresponding boundaries due to zoning and scale aggregation issues imposed by the well documented Modifiable Areal Unit Problem [8]. Resolving these challenges requires the definition of a zoning and aggregation scheme that can be clearly justified for the urban function under study.

Urban mobility is one such urban function for which the definition of better neighborhood boundaries would have broad impacts. The patterns of the movement of people through the built environment impact many types of urban functions. Transportation infrastructure is the most pronounced example [9]. Less obvious are the secondary impacts on, for example, energy systems, which manifest through the daily cycle of neighborhood-scale building occupancy. Moreover, these patterns of movement are also rough approximations of *social interaction patterns* [10,11]. In addition to the naturally anticipated ramifications for social infrastructure (e.g., schools, parks, libraries, grocery stores, and so forth), the power law scaling relationships that many fundamental indicators of urban activity demonstrate with city population (including GDP, patents, and crime) have theoretically predicted dependencies on social interaction patterns [2,12–14]. Thus, defining zones that can be justified based on human mobility patterns would have value for understanding a wide variety of urban functions and informing the design of relevant services or infrastructure.

Neighborhoods have a long theoretical history of being qualitatively defined by barriers or breaks in pedestrian mobility and associated social connectivity [15,16]. Such barriers are described as being defined generically by open spaces such as transportation infrastructure, parks, water bodies, and so forth, many of which describe the rights-of-way (ROWs) of past infrastructure interventions [17,18]. Recent work on so-called *Community Severance* has quantified the barrier impacts of the width of transportation infrastructure in particular, while also suggesting that these findings may hold for the other types of barriers [19]. However, there is limited work using a static width heuristic (i.e., the measured width of large barriers) to define neighborhoods, particularly in an automated fashion.

Recent work on what have been termed *sanctuary areas* provides a potential method for defining neighborhoods using the community severance heuristic. Modernist planners in the 1960s designed neighborhood units explicitly bounded by wide roads [20]. Thus, researchers have attempted to extract such regions by identifying urban areas bounded by roads of regional topological importance [21]. Interestingly, even pre-1960s intra-urban areas identified with these tools have found use in some urban morphology classification tasks [22]. This finding is likely partially explained by community severance principles and that the topological importance of individual roads correlates with their barrier width. However, this relationship—between topological importance and width—is not guaranteed, and thus a framework that uses barrier width directly, while also allowing the capture of other non-road barriers (e.g., parks), theoretically will have more utility. Using a width heuristic in characterizing neighborhood boundary barriers also provides additional benefits. In particular, it allows for the definition of a hierarchy of nested neighborhoods parameterized by the barrier width, with larger barriers defining geographically larger neighborhoods. This notion provides a potential compliment to studies aimed at detecting hierarchical structure at the super-urban scale, generally through an analysis of the fractal dimension of clusters [23–25]. Moreover, this hierarchy provides a range of scales to test for structural importance as opposed to an *a priori* assumption of a single important user-defined scale.

Here, we develop an automated method for extracting neighborhood structure based on readily available features of a city’s physical form: building parcels. Specifically, we are interested in uncovering neighborhood substructures within a city and determining the natural scale upon which such neighborhoods form, where scale refers to the spatial extent of the neighborhoods and barriers that define them. We compare the scales that emerge from this analysis across three American cities (San Francisco, New York, and Houston) with varying topography, transit modalities, and urban planning regimes. To do so, we use concepts from the topological data analysis method of persistent homology. These methods allow us to identify characteristic road and public-space scales in cities and to robustly distinguish them from the noise. Comparing the locations and numbers of these scales across cities reveals a lack of consistency in morphology even between cities that superficially appear to be similarly gridded. Given the aforementioned relationship between neighborhoods and infrastructure service provisioning within cities, our work also represents a significant step toward a more nuanced understanding of how physical urban morphology shapes energy and mobility systems.

## Results

### Uncovering neighborhood structure

For the purposes of introducing a general set of methods, we define *barriers* here as the negative space between places of interest—more specifically, between parcels. A parcel represents the outer bounds of a given piece of (landed) property. Only parcels that correspond to buildings are included; we exclude rail and road infrastructure, parking lots, parks, and other similar open spaces. We collected parcels for three U.S. cities: San Francisco, Houston (the “urban zone” within the I-610 loop), and the New York Borough of Manhattan [26]. These three cities were chosen because they comprise a relatively diverse set of American geographies (east coast, west coast, south), regulatory regimes, and periods of development. While much of Manhattan was planned and built before the advent of the automobile, large sections of both San Francisco and Houston were built (or re-built, in the case of San Francisco) afterwards. Additionally, a significant portion of Houston was developed post World War II and the advent of the interstate freeway system [27]. While Manhattan and San Francisco were also non-trivially impacted by this new infrastructure, both cities saw widespread, and successful, opposition to most planned freeways of the mid-20^th^ century [28,29]. As a result, freeways—generally significant barriers—are more prominent in the urban landscape of Houston as compared to the other two cities. In addition, Houston is the only major city in the United States without Euclidean zoning laws dictating parcel land use. This, however, has resulted in a greater emphasis on street and thoroughfare plans, and thus relatively wide streets by American standards [26].

Identifying nested neighborhoods in this context is fundamentally a hierarchical clustering problem, where identified clusters represent neighborhoods. Here, we use single linkage clustering to directly parametrize the barrier width heuristic as a threshold ϵ (see Methods for more details). Note that more complex barrier measurements could be captured with the same parameter. As a test of the method and to develop intuition, we first apply this framework to a synthetic example. Results from this model problem demonstrate that our framework both uncovers neighborhoods consistent with the notion of barriers and is robust to the noise that is unavoidably present in real city building parcel data due to, for example, errors or inconsistencies in public record-keeping (see S1 File for an extended discussion of the effects of noise on our model). Next, we apply our framework to empirical data from our three chosen cities and find that the neighborhood sub-structure we uncover in this objective, automated fashion is consistent with the notions of barriers or edges described in the urban planning literature. For example, we find that highways in particular behave as physical barriers that drive the partitioning of neighborhoods as described in seminal work by Lynch in the early 1960s [15].

**Fig 1:**
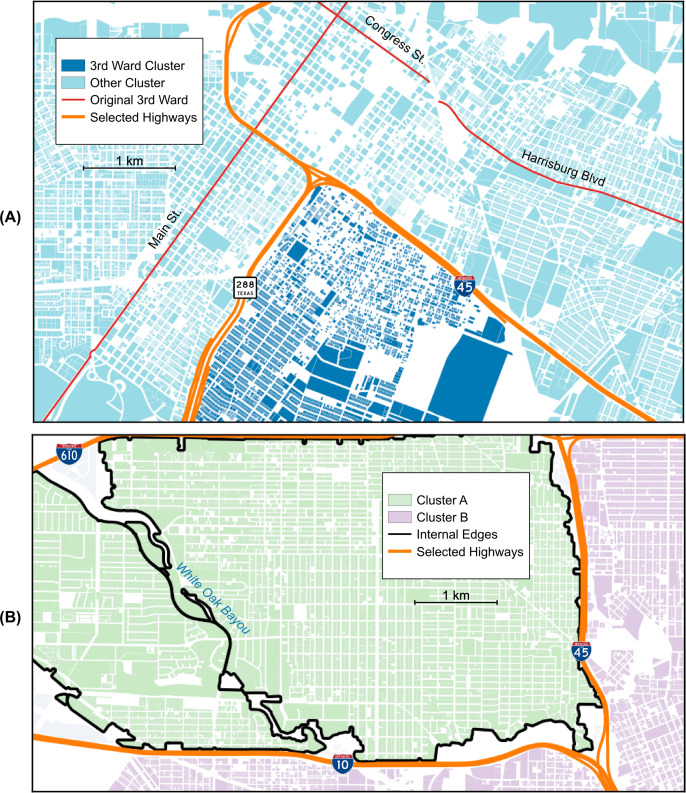
(a) The Third Ward, indicated in dark blue, as described by our method. Modern definitions for the northeast and northwest borders are the highways shown in orange; the original political boundaries are shown in red. (b) Neighborhood separated from the rest of Houston by the Katy Freeway (and I-45) in the northwest corner of the Houston I-610 loop (light blue and grey). This neighborhood can be further subdivided by our method into two neighborhoods roughly corresponding to the Greater Heights and Lazybrook/Timbergrove “super neighborhoods” as defined by the city government (black lines).

These barriers are particularly evident in Houston with its many wide highways that cut through the urban core. The Katy Freeway (I-10) northwest of downtown Houston is popularly known as an example of a particularly wide highway [30]. Our method identifies the neighborhood to the north of this highway as one of the more isolated large sections of the city within the loop (Fig 1B). However, we find that the most isolated neighborhood in the 610 Loop is the Third Ward (Fig 1A). This particular neighborhood was systematically enclosed by I-45 and State Highway 288 over the period between the late 1960s and the early 1980s. The construction of these highways led to an exodus of wealthier residents and community decline in the latter half of the twentieth century [31]. This change in local character subsequently changed perceptions of the boundaries of the Third Ward. The original political definition included much of what is now Downtown, with borders at Main Street to the northwest as well as Congress Street and Harrisburg Boulevard to the northeast [32]. However, more modern definitions of the Third Ward now often define SH 288 and I-45 as the northwest and northeast borders, respectively [33]; these are the exact same boundaries that we find using our method.

Recovering these shifting neighborhood boundaries in an objective fashion without using any domain knowledge is valuable and demonstrates the complexity of the information embedded within the urban form. Additionally, this result can be seen as a validation of the ideas behind our method that then gives us confidence to use it to uncover features of the urban landscape that are not *a priori* known. Since our technique is by construction hierarchical (see Methods), we can use it to identify a nested sequence of barrier-defined neighborhoods. Each neighborhood in this sequence will be bounded by similar types of edges; however, these edges will increase in width as we move higher in the hierarchy. We interpret the width of the bounding edges as a proxy for the isolation of one neighborhood from its neighbor; thus, the distinction between two neighborhoods at a higher level in the hierarchy is larger than two neighborhoods lower. For example, consider the neighborhood north of I-10 described earlier. This neighborhood can be further subdivided in two along the White Oak Bayou. These subdivisions roughly correspond with the Greater Heights neighborhood to the east and Lazybrook/Timbergrove neighborhood to the west [34,35].

### Scales of neighborhood partition

Now that we have demonstrated that our techniques can reasonably identify neighborhood substructure, both individually and hierarchically, we can use them to investigate urban morphology more generally. In particular, we aim to characterize the phenomenon of neighborhood partitioning globally over entire cities. To do so, we first model the hierarchical subdivision of neighborhoods by barriers of specific widths as a Markov chain. The state space for this process is the set of all possible barrier widths {0,1,…,*max*_*c*_(*B*_*c*_)}, where *B*_*c*_ refers to the width at which neighborhood *c* is born (i.e., is first identified in our single-linkage clustering method). Thus, the analog of time in this stochastic process is the level in the dendrogram, proceeding from the birth of a parent cluster to the birth of a child cluster. In this context, the transition probabilities *P*_*ij*_ of the stochastic process refer to the *subdivision probability* that a neighborhood bounded by a barrier of width *i* will be subdivided by a barrier of width *j*, where *i* > *j*. It is reasonable to treat this stochastic process as Markovian because there is no *a priori* reason to expect that neighborhoods of some scale are always subdivided in the same way.

These values allow us to study the relationship between bounding and subdividing edges, as well as the most disproportionate widths of barriers that subdivide neighborhoods. Note that much of the activity in the subdivision probability matrix **P** is likely to be near the diagonal, reflective of imperfections in the structure of the city (e.g., a grid where one street was platted slightly narrower). Thus, entries farther from the diagonal are more indicative of interesting structure in the city. Barrier widths that subdivide a variety of larger neighborhoods will see multiple of these entries in their respective column. Since **P** is row stochastic but not column stochastic [36], the sum over the columns ∑_*i*_*P*_*ij*_ captures these deviations. In particular, ∑_*i*_*P*_*ij*_ is a vector representing how disproportionately all barrier widths subdivide neighborhoods across the entire city. We term this quantity the *disproportionality vector*.

Although this measure describes which barrier widths are *globally* (that is, across all widths) disproportionate, it does not provide a description of which widths characterize continuous *local* regions of high disproportionality. However, these characteristically disproportionate widths are very meaningful, as they highlight different width regimes that do not exist solely due to noise in the data. To isolate these characteristic barrier widths, we turn to the concept of persistence from topological data analysis. Persistence is analogous to the topographic prominence problem of identifying which peaks characterize mountains, though generalized to local maxima (or minima) across all functions [37]. We apply this idea to the disproportionality vector and extract the most persistent barrier widths (see Methods for more details). These values are labeled as *characteristically disproportionate widths*.

**Fig 2:**
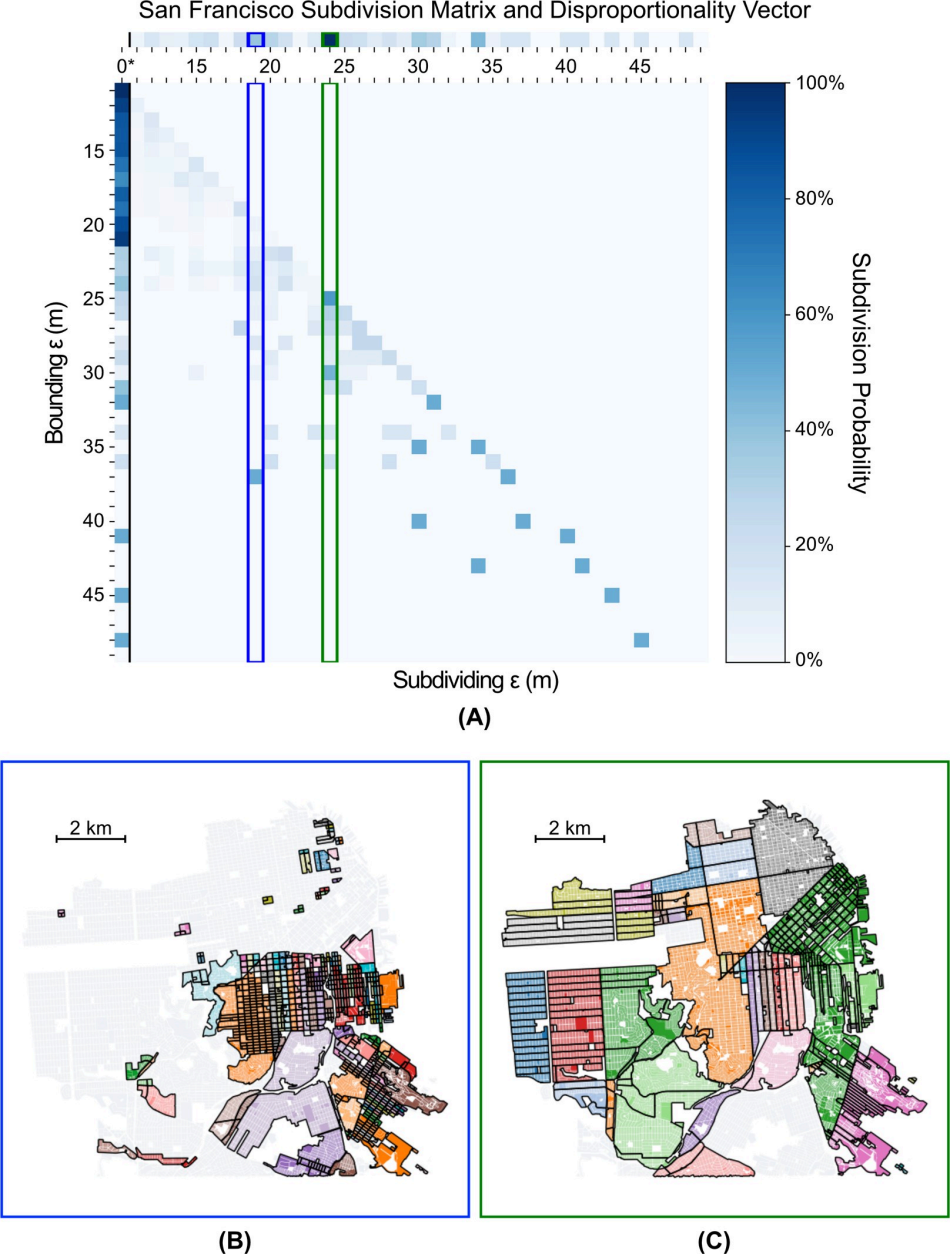
(a) Subdivision Matrix for San Francisco, with subdivisions by top three characteristically disproportionate widths (see methods): (b) 19m, 34m, and (c) 24m highlighted. Each cell in the matrix represents the probability that the next interior divider for a cluster bounded by a road of width *i* has width *j* (where i is represented by rows and *j* is represented by columns). The first column (0*) in this matrix is the sum total for any edge of width less than 11m to accommodate noise introduced by data downsampling. See Methods for more details. The distribution at the top is the sum of these probabilities row wise—the disproportionality vector—showing internal widths that disproportionately subdivide neighborhoods across the entire city.

**Fig 3:**
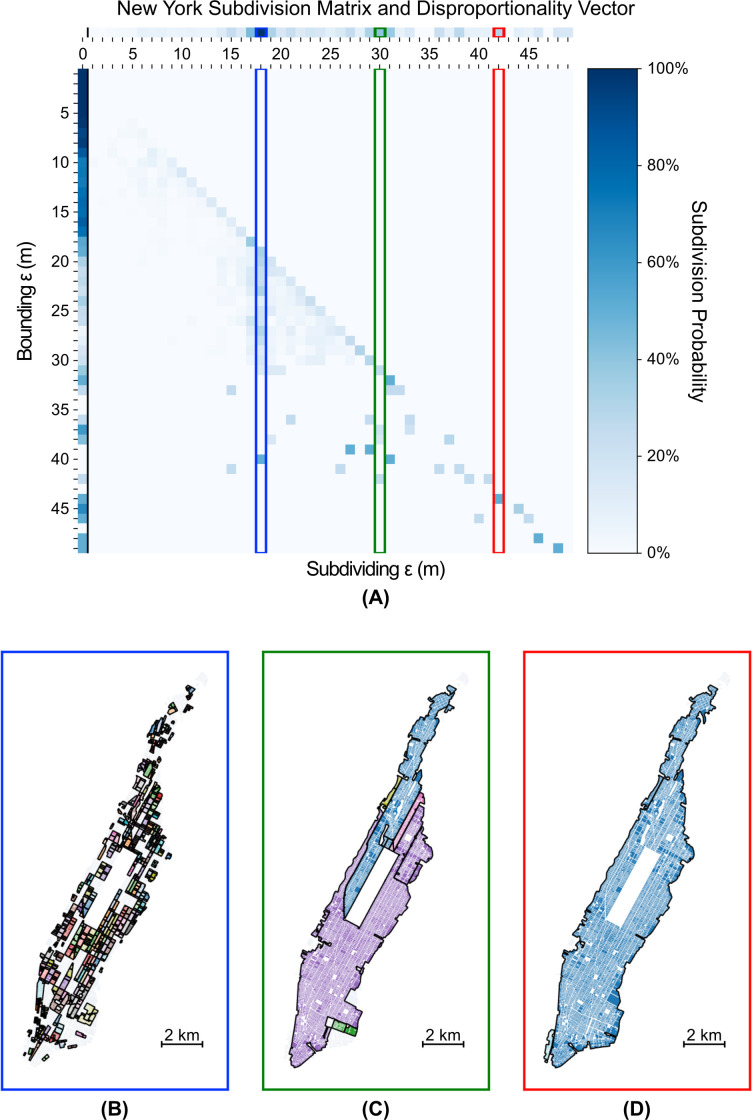
(a) Subdivision Matrix for New York, with top three characteristically disproportionate widths of (b) 18m, (c) 30m, and (d) 42m highlighted. The bottom portion of the figure depicts the clusters (solid color) with primary internal edges of width 18m, 30m, or 42m (internal black lines).

**Fig 4:**
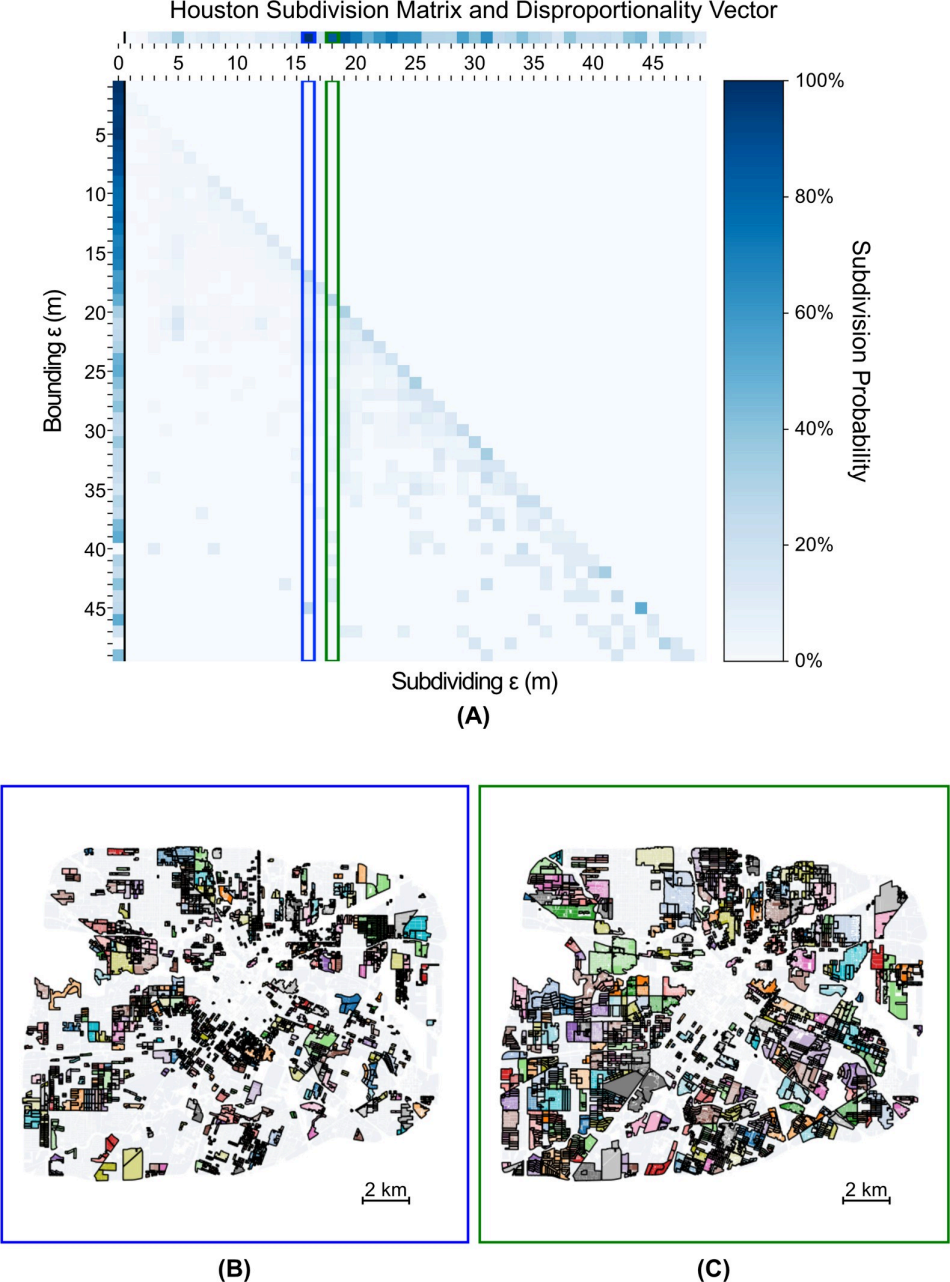
(a) Subdivision Matrix for Houston, with top two (sub 50m) characteristically disproportionate widths of (b) 16m, and (c) 18m highlighted. The bottom portion of the figure depicts the clusters (solid color) with primary internal edges of width 16m or 18m (internal black lines). Note that the disproportionality vector appears spread over a larger set of values (both smaller and larger) than New York and San Francisco.

We plot the subdivision probability matrices, disproportionality vectors, and characteristically disproportionate widths for San Francisco, Manhattan, and the core portion of Houston (inside the 610 freeway loop) in Figs 2A, 3A and 4A. We find that although each city has distinct scales at which neighborhoods form, a potentially universal scale of 18–19 m appears across all three cities. This similarity indicates that despite the significantly divergent land-use and urban planning policies of San Francisco and New York (centralized) versus Houston (decentralized), neighborhoods tend to be severed by, or, equivalently, form around, barriers of similar width. This potentially universal scale of neighborhood development is likely an artefact of the typical width of a U.S. car lane of 2.7–3.6m [38]. Thus, a typical two to three lane road with parking and pedestrian facilities on both sides would approach ~18m in width. The existence of this potentially universal scale suggests that, structurally, there is a different regime of neighborhood partition around barriers larger than 18-19m than below or at that threshold. This regime change indicates that any barrier (e.g., a roadway, railway, or park) wider than 18-19m causes relatively more physical isolation between subregions in all three cities than is typical, and thus drives the emergence of neighborhoods.

Beyond this single potentially universal scale, however, neighborhood partitioning begins to diverge between the three cities, with each city’s larger scales being driven by its own local features. For San Francisco, we find that neighborhoods also form at scales of 24m and 30m. Fig 2C shows the neighborhoods that have primary internal edges of width 24m. Several intriguing and interpretable features are apparent at this scale. Throughout the Mission District, for example, we find that these internal barriers tend to be east-west streets. The surrounding edges, however, tend to be north-south streets, perhaps reflecting the larger gradations in “neighborhood feel” when traveling east to west. 24m stands out as the second significant scale in subdivision probability across all neighborhoods bounded by wider edges. The primary pattern of north-south corridors in the Mission District is again apparent at this scale. Similarly, we find that the Richmond and Inner/Outer Sunset Districts also have well defined east-west corridors, albeit with narrower edges dividing them. The different morphologies between the neighborhoods north and south of Market St are also well highlighted (with South of Market being severed uniformly by the wider 24m roads) by this analysis, reflecting the historical development patterns of the city [39].

We find that Houston (Fig 4) has neighborhoods that form at a smaller scale (15 m) than those in either New York or San Francisco. Moreover, the neighborhoods formed at this scale lie near the periphery of the city, in contrast with the other two cities whose neighborhoods that form at smaller scales tend to lie near their older, central sections. This result is somewhat surprising given Houston’s strong preference towards the private automobile for travel [40], which might imply that wider, car-oriented streets ought to dominate across the entire city. Comparing the disproportionality vectors for Houston and New York directly, we find that although Houston tends to have neighborhood partitions disproportionately occur at larger scales (due the preponderance of wider barriers), it does indeed begin at smaller scales than New York. New York, on the other hand, sees more of its neighborhood partitions occur in a small range between 17m and 18m. This result suggests that although Houston, across the entire city, has larger divisions than Manhattan, the smallest scales of neighborhoods are more closely tied together and isolated than in Manhattan. Similar patterns emerge when comparing San Francisco and Houston. In general, Houston appears to exhibit more heterogenous neighborhood partitioning, perhaps reflecting its distinct planning ethos.

## Discussion

We have introduced an automated, objective method to construct a multi-scale hierarchy of neighborhoods in cities. These neighborhoods, constructed to follow natural breaks in urban mobility through an application of *community severance* theory, have potential applications in areas ranging from transportation modeling to studies of urban allometry. This method also allows us to extract the most characteristically disproportionate scales for individual cities. Our method uses only simple data—the shape and locations of buildings in space—that is readily obtainable, potentially even from publicly available satellite imaging [41]. From this information, we build a dendrogram from which the important scales can be found by calculating subdivision probabilities.

We applied this method to three US cities with distinct geographic histories and planning regimes. We find that many known neighborhood boundaries, such as highways, railroads, and water bodies, are well described by the neighborhood hierarchy, as expected. Similarly, the regions adjacent to these boundaries that are identified as neighborhoods roughly correspond to traditional definitions that rely on a significant amount of specific local knowledge. Despite the differences between these three cities, we consistently find the most disproportionate scale to be 18-19m. This points to some latent feature that consistently drives neighborhood partitioning, at least in the United States. However, we find that the range of scales that divides neighborhoods is far more diverse in Houston than in New York or San Francisco. In particular, we note that Houston has characteristically disproportionate scales both smaller and large than those found in New York, perhaps implying that Houston is more closely connected locally but more divided at larger scales. Both of these patterns demonstrate how the method we have introduced provides a succinct, readily comparable way to analyze neighborhood partitions across scales for individual cities.

These results clearly point to future extensions of the method we have presented. In particular, it naturally can be used to conduct a similar analysis for additional cities across the United States. This would allow us to measure the extent to which the common scale we have found here is universal across all American cities, and to develop a better understanding of the underlying latent features that drive such universal scales. Similarly, future work could examine which city cores, if any, tend to be heterogeneously divided like Houston, as opposed to uniformly divided like San Francisco or New York. These extensions of our work could further inspect the relationships between the morphological features uncovered by this method and historical narratives of individual cities. In particular, further work could seek to understand how the characteristic scales uncovered by this method differ across neighborhoods of different ages of construction. Such historical contextualization would greatly aid the interpretation and use of the method’s results for planning in local contexts.

Our methods also allow for the use of bespoke distance metrics that may capture different barrier effects. Future work could include distance metrics with, for example, embedded topographic elevation or bike lane network topology. Finally, future work is needed to understand how our results generalize to cities around the world, especially those that do not have similar histories of gridded development or centralized planning (e.g., informal settlements). The simplicity of the required input data is ideal for this purpose. The rise of remote sensing imaging and deep learning is making it possible to capture building footprints anywhere in the world [41]. Thus, our proposed methods will be relatively easy to extend even to areas with limited traditional government datasets.

Finally, our proposed methods open the door for further studies testing critical hypothesis on the impact urban-scale physical structures have on the dynamics of urban functions, from mobility to social networks. For example, one could use the neighborhoods we have identified here to study the effect of severing boundaries on urban-scale mobility patterns. The relationships between these barrier defined neighborhoods and the flow-hierarchy defined in [42] is particularly interesting. Alternatively, one could measure the morphology of social interactions within and across different neighborhoods—potentially providing grounding for assumptions made in urban-scaling research.

## Methods

### Data

We collected parcel data for Houston, San Francisco, and New York City. The Houston and New York data were subset to smaller geographic units: the I-610 loop in Houston and Manhattan in New York. Outlying islands for San Francisco and Manhattan were also removed. The remaining parcels were subset to those that represent buildings, removing rail and road infrastructure, parking lots, parks, and other similar open spaces. This process left 148,353 parcels for San Francisco, 39,736 parcels for New York City, and 149,434 parcels for Houston. To aid our analysis and interpretation, the geometries for all three cities were re-projected into Euclidean space using the corresponding Universal Transverse Mercator (UTM) zone coordinate system. The resulting parcels in San Francisco were down-sampled to points along the perimeter 4 meters apart to improve computation time.

Raw data are available from the corresponding local government websites and updated regularly. Processed data, including geometries used to subset the data and code, are available at https://github.com/Urban-Informatics-Lab/auto-urban-substructure-ident [43–49].

### Single linkage clustering

In Single Linkage Clustering, a subset of objects are defined to belong to the same cluster if they are within a distance *ϵ* from each other. At *ϵ* = 0, each object belongs to a cluster than only include objects immediately adjacent. As *ϵ* increases, the number of clusters decreases until all objects belong to the same cluster. See [50] for implementation details. In the context of this research, clusters represent neighborhoods. This clustering process is equivalent to particular filtrations of simplices in the persistent homology literature, from which we borrow some terminology. The clusters produced by this algorithm are equivalent to the 0 dimensional homology classes in a Vietoris-Rips or Cech complex for equivalent parameter *ϵ* [51]. All homology classes are characterized by a *birth* and *death ϵ* corresponding to the smallest and largest values at which the class exists, respectively [37,51,52]. However, we have one small difference in terminology. In the persistent homology literature, all (0-dim) homology classes are born at *ϵ* = 0, and, at a merge, only one of the classes is marked as “dying.” Here, however, all clusters in a merge have the corresponding *ϵ* associated as their death. The same *ϵ* becomes the birth for a new cluster.

The objects in our metric space are parcel geometries. The distance metric is the Euclidean distance between two parcels, discretized to the integers for ease of computation. The main benefit of applying Single Linkage Clustering on this particular metric space is ease of interpretability. The *ϵ* at which two clusters of parcels merge (and a new cluster is born) is a measurement of the width of the negative space between them. As negative space in this dataset is reflective of barriers by virtue of not including roads or open space, the birth and death values of an individual cluster represent the width of barriers that, respectively, subdivide and bound it.

### Persistence

Persistence is closely related to the mountaineering concept of topographic prominence. Topographic prominence attempts to answer the question “what is a mountain?” with the understanding that not every single local maximum (of elevation in this case) qualifies the peak as its own mountain. A mountain, such as Everest, may have two or more summits, one of which is considered prominent—and defines the height of the mountain—and one that is not. This prominence is measured as how far one has to descend from one peak before beginning the ascent on a taller peak [37].

In Topological Data Analysis, topographic prominence is analogous to persistence, as anticipated earlier, and is measured similarly. A primary context for its use is persistence-based clustering, in which the mountains in the previous example are analogous to clusters organized around basins (or hills) of attraction [53]. Persistence-based clustering works by applying an algorithm parametrized by the persistence threshold τ twice for two different values of the parameter. When τ = ∞, this algorithm produces a persistence diagram depicting the birth (in this context, the value of the maximum) and death (the lowest value between the local maximum and the highest adjacent maximum) for each local maximum. Typically, births are plotted on the horizontal axis while deaths are plotted on the vertical axis; in this context, the persistence of each maximum is encoded in its y-distance above the line *y* = *x*. This diagram can then be used to identify τ for the second pass of the algorithm, which identifies clusters with local maxima that persist longer than τ. We use this clustering technique to identify persistent clusters in the disproportionality vector, with the local maxima of the clusters identified as the characteristic disproportionate widths.

## Supporting information

S1 File Supporting information for automated identification of urban substructure for comparative analysis. (PDF)
