# Supplementary material for: Automated identification of urban substructure for comparative analysis
Source: PLoS One. 2021 Jan 14;16(1):e0245067. doi: 10.1371/journal.pone.0245067 (PMC7808605; doi:10.1371/journal.pone.0245067)
Supplement: S1 File — (PDF) [file pone.0245067.s001.pdf]

S1 File. Supporting Information for Automated identification of urban substructure for comparative analysis

This Supplemental Information serves to provide further details on the robustness and elucidate the information encoded by dendrograms produced by single linkage clustering. To do so, we create a series of synthetic city models with a hierarchical street pattern and apply our methods to these synthetic examples. As demonstrated in Fig 1a, we generate a series of polygons representing parcels that, in aggregate, take the form of overlapping street grids with differing road widths. The included example data consist of two grids of different morphology. The left grid of sixteen blocks has a hierarchical street pattern, with four sets of four blocks severed from each other by a horizontal and vertical road, each 30m wide. The four blocks within each set are similarly divided by roads of 15m. The grid on the right of twelve blocks is simpler, with all five roads being 24m in width. The two grids are themselves severed by a wider road 50m in width.

Single Linkage Clustering is applied to this city model as detailed in Methods. As some parcels in the synthetic data are directly adjacent, the first set of neighborhoods appears at $\epsilon = 0$m. This can be seen at the base of the dendrogram in Fig 1b. Intuitively, these initial neighborhoods represent blocks. Moving up the diagram, the next set of merges for the grid on the left appear at $\epsilon = 15$m. This directly corresponds to the width of the narrower barriers in that grid. Similarly, at $\epsilon = 24$m, all of the blocks in the right grid merge into a single cluster, delineating that portion of the synthetic city as its own neighborhood. The neighborhoods resulting from merges at 15m and 24m are shown in Fig 1a. The left grid has a third merge corresponding to the wider internal barriers at 30m, at which the left grid is viewed as a unique neighborhood. The two remaining neighborhoods are severed from each other by a barrier 50m in width—the dendrogram records this as the final merge at $\epsilon = 50$m. As demonstrated by the dendrogram in Fig 1b, Single Linkage Clustering neatly captures the hierarchy of neighborhoods implied by the Community Severance heuristic that wider barriers are more severing. It simultaneously describes the isolation of each recovered neighborhood.

As described in Scales of Neighborhood Partition, we summarize the information captured by this clustering method by modeling the hierarchical subdivision visible in the dendrogram as a Markov chain. The state space of this Markov chain can be visually understood as the horizontal levels present in Fig 1b. We plot the resulting *subdivision* (transition) *probability* matrix and *disproportionality vector* for the synthetic example

in Fig 2. In this perfectly clean example, the three persistent values of disproportionality vector—the *characteristically disproportionate widths*—capture the partitioning barrier widths of each grid (15m, 24m, and 30m) exactly.

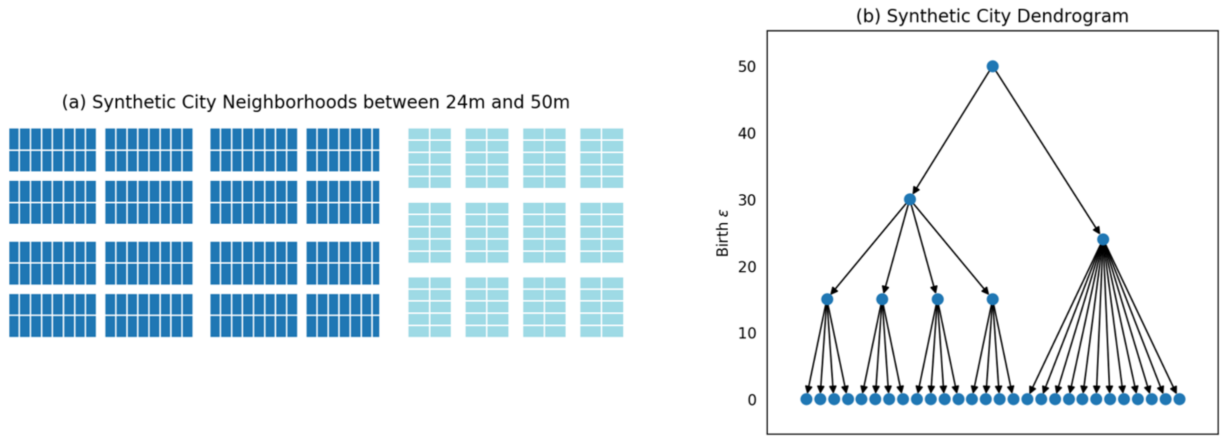

Figure 1: Synthetic city and dendrogram. (a) depicts the parcels of the synthetic city grouped by neighborhoods that appear between 24m and 50m. (b) depicts the dendrogram resulting from single linkage clustering on the parcels. Note: Two distinct structures are visible in the dendrogram—these directly reflect the two different grids present in the synthetic city.

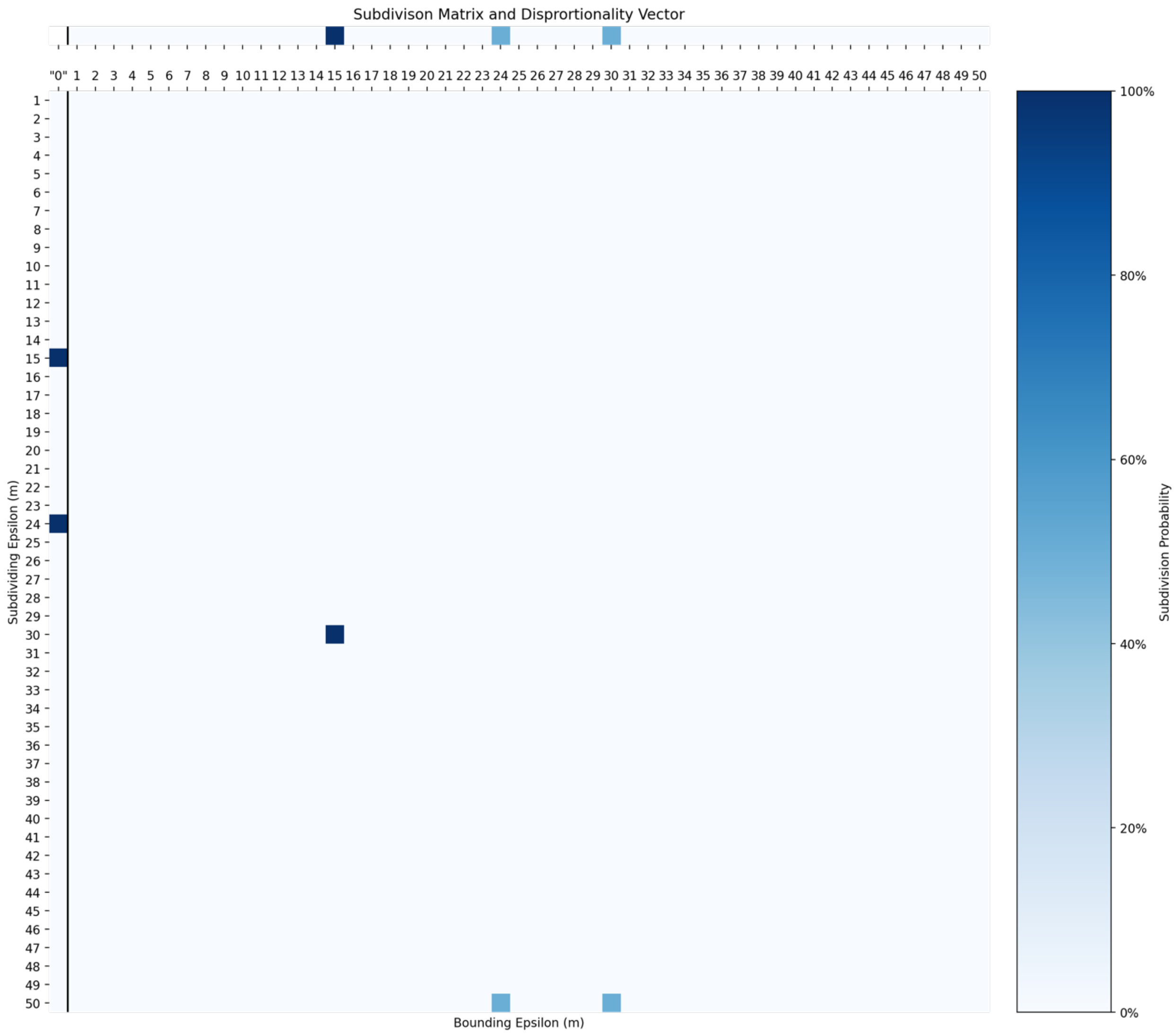

Figure 2: Subdivision matrix and disproportionality vector for synthetic city. The 15m, 24m, and 30m barriers that partition the two grids are clearly highlighted in the vector.

In Figs 3 and 4 we demonstrate the same methodology, from clustering to characteristically disproportionate widths, on a version of the same synthetic example with added noise. We do this to demonstrate how noise might affect the presented methodology; however, we note that such changes will depend on the details of how noise is modeled. Here, we model noise by scaling each edge of each block away from the opposite edge by a factor C, where $C \sim \mathcal{N}(1, 0.01)$. Note that while this does disrupt the exact organization of the dendrogram in Fig 3b, distinct horizontal levels are still present, if smeared along ϵ. This is reflected in the three sets of values—corresponding to the 15m, 24m, and 30m scales of the clean synthetic example—in the disproportionality vector of Fig 4. We find, from an application of the persistence methodology described in Methods, that there are now four characteristically disproportionate widths: 17m, 23m, 30m, and

14m. While these do not exactly match the latent parameters of the synthetic example, they do capture a similar substructure of the neighborhoods. Moreover, they do accurately summarize the dendrogram for the noisy synthetic example.

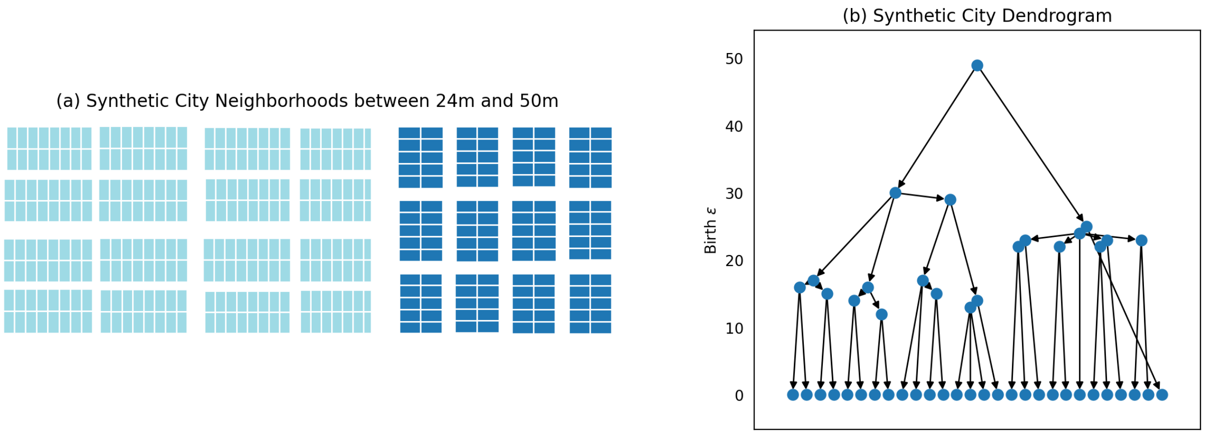

Figure 3: Synthetic city and corresponding dendrogram after noise has been introduced. (a) depicts the parcels of the synthetic city grouped by neighborhoods that appear between 24m and 50m. (b) depicts the dendrogram resulting from single linkage clustering on the parcels. Note that the dendrogram maintains horizontal structures around 15m, 24m, and 30m.

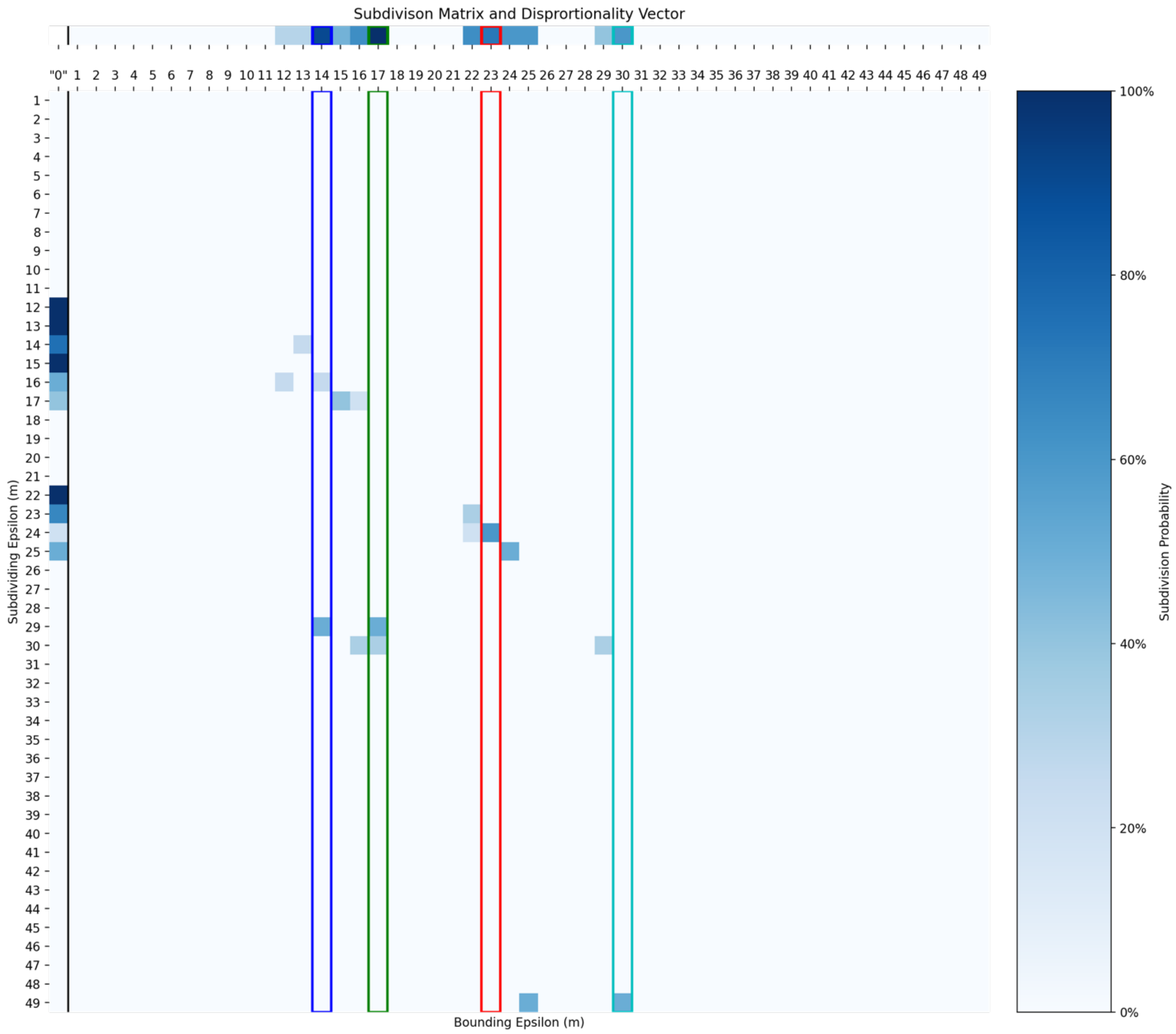

Figure 4: Subdivision matrix and disproportionality matrix for synthetic city after noise has been introduced. There are four characteristically disproportionate widths highlighted—17m, 23m, 30m, and 14m—that closely correspond to the values before the introduction of noise.
